# Correlation of [^18^F]florbetaben textural features and age of onset of Alzheimer’s disease: a principal components analysis approach

**DOI:** 10.1186/s13550-021-00774-x

**Published:** 2021-04-21

**Authors:** Jing Li, Emanuele Antonecchia, Marco Camerlenghi, Agostino Chiaravalloti, Qian Chu, Alfonso Di Costanzo, Zhen Li, Lin Wan, Xiangsong Zhang, Nicola D’Ascenzo, Orazio Schillaci, Qingguo Xie

**Affiliations:** 1 Department of Biomedical Engineering, Huazhong University of Science and Technology, Luoyu Road, Wuhan, 430074 China; 2 Department of Medical Physics and Engineering, Istituto Neurologico Mediterraneo NEUROMED I.R.C.C.S, Via Dell’Elettronica, 83008 Pozzilli, Italy; 3 Department of Biomedicine and Prevention, University of Tor Vergata, 86100 Rome, Italy; 4 Tongji Medical College, Huazhong University of Science and Technology, Hangkong Road, Wuhan, 430030 China; 5 Department of Oncology, Tongji Hospital, Jiefang Avenue, Wuhan, 430030 China; 6 Department of Radiology, Tongji Hospital, Jiefang Avenue, Wuhan, 430030 China; 7 Department of Software Engineering, Huazhong University of Science and Technology, Luoyu Road, Wuhan, 430074 China; 8 The First Affiliated Hospital, Sun Yat-sen University, Zhongshan 2nd Road, Guangzhou, 510080 China; 9 NIM Competence Center for Digital Healthcare GmbH, Potsdamerplatz, 10, 10785 Berlin, Germany; 10 Universita degli Studi del Molise, Via Francesco de Sanctis, 1, 10115 Campobasso, Italy

**Keywords:** Early-onset Alzheimer’s disease, Textural analysis, Positron emission tomography

## Abstract

**Background:**

When Alzheimer’s disease (AD) is occurring at an early onset before 65 years old, its clinical course is generally more aggressive than in the case of a late onset. We aim at identifying [$^{18}$F]florbetaben PET biomarkers sensitive to differences between early-onset Alzheimer’s disease (EOAD) and late-onset Alzheimer’s disease (LOAD). We conducted [$^{18}$F]florbetaben PET/CT scans of 43 newly diagnosed AD subjects. We calculated 93 textural parameters for each of the 83 Hammers areas. We identified 41 independent principal components for each brain region, and we studied their Spearman correlation with the age of AD onset, by taking into account multiple comparison corrections. Finally, we calculated the probability that EOAD and LOAD patients have different amyloid-$\beta$ ($A\beta$) deposition by comparing the mean and the variance of the significant principal components obtained in the two groups with a 2-tailed Student’s *t*-test.

**Results:**

We found that four principal components exhibit a significant correlation at a 95% confidence level with the age of onset in the left lateral part of the anterior temporal lobe, the right anterior orbital gyrus of the frontal lobe, the right lateral orbital gyrus of the frontal lobe and the left anterior part of the superior temporal gyrus. The data are consistent with the hypothesis that EOAD patients have a significantly different [$^{18}$F]florbetaben uptake than LOAD patients in those four brain regions.

**Conclusions:**

Early-onset AD implies a very irregular pattern of $A\beta$ deposition. The authors suggest that the identified textural features can be used as quantitative biomarkers for the diagnosis and characterization of EOAD patients.

## Background

Alzheimer’s disease (AD) is a neurodegenerative disorder characterized by a progressive cognitive decline and dementia [1, 2]. The occurrence rate of such a diagnostic scenario is approximately doubling every 5 years after the age of 65 years old. Such a state, which represents the majority of patients, is called late-onset AD (LOAD). However, in the case of an early onset of the AD before an age of 65 years old (EOAD), the clinical course is more aggressive than in LOAD patients [3–9]. Clinical features of AD are characterized by the impairment of cognitive functions leading to a progressive loss of the autonomy of patients, especially in the daily activities [10]. The correct identification of the disease is crucial to distinguish AD from other types of dementia and to use the correct therapeutic approach [11, 12]. In the last decade, the role of amyloid $\beta$($A\beta$)- and tau-mediated pathology in the development of the disease has been stressed. Currently, the most promising approaches involve, on the one hand, the detection of soluble biomarkers in the cerebrospinal fluid and, on the other hand, the molecular imaging of glucose metabolism, $A\beta$ and tau accumulation in the brain cortex with positron emission tomography (PET). Glucose metabolism shows typical alterations in AD, which are significantly related to the accumulation of tau and $A\beta$ [13, 14]. However, EOAD introduces an additional element of complication in the AD landscape. While differences in cerebral metabolic impairment between EAOD and LOAD were observed and also supported by histopathological findings [15, 16], suggesting the existence of biological subtypes of AD, the $A\beta$ deposition seems not to be correlated with the age of onset of AD [17].

One possibility, which so far has been explored only theoretically, is to find heterogeneity patterns of the $A\beta$ deposition, which may significantly depend on the age of the AD onset. Textural analysis is an emerging technique for the quantitative study of tracer uptake in different regions of the brain. For example, in neuropsychiatric disorders, textural differences were found between the autism spectrum disorder and control groups in the right hippocampus, left choroid-plexus, corpus callosum and cerebellar white matter [18]. Another recent study indicated that cerebral morphometric alterations allow discrimination between the patients with attention deficit hyperactivity disorder and control subjects [19]. As the three-dimensional radiomic features quantify localized heterogeneities in brain morphology and functionality, they can be associated with neurodegenerative phenomena. By way of example, corpus callosum textures were considered as magnetic resonance imaging (MRI)-based biomarkers of AD [20]. Recently, it has been found that textural features have a distinct ability to classify AD versus healthy control (HC), mild cognitive impairment (MCI) versus HC, and AD versus MCI with maximum average accuracies of 91.5%, 83.1%, and 85.9%, respectively [21]. On this basis, biomarkers have been identified in 2-deoxy-2-[$^{18}$F]fluoro-d-glucose (2-[$^{18}$F]FDG) PET by distinguishing AD from MCI in brain regions mainly distributed in the temporal, occipital and frontal areas [22]. However, textural-based analysis of the $A\beta$ deposition for the classification of EOAD and LOAD has not been reported yet.

It cannot be expected that the difference of the $A\beta$ deposition between EOAD and LOAD can be identified in the entire cortex. In fact, it was observed that EOAD patients exhibit a higher degree of cortical atrophy and reduced perfusion and metabolism in parietal and lateral temporal cortices with respect to LOAD patients [14, 23, 24]. Although no evidence regarding the $A\beta$ deposition has been found in these regions [17], it has been not considered until now that the heterogeneity of the accumulation of plaques may present also signs of the structural decline of AD brain and may differ between EOAD and LOAD patients. As a result, a reliable textural indicator of EOAD is currently missing and a texture-based analysis of the difference between EOAD and LOAD patients is not reported in the literature.

In this paper, we study 93 textural features defining the patterns of the $A\beta$ deposition extracted from [$^{18}$F]florbetaben PET in 83 Hammers [25] regions covering the entire brain. By analyzing the correlations between the principal components extracted from each brain region and the age of onset of AD in a sample of 43 patients with AD diagnosis, we aim at identifying the brain areas that can express significant differences in the $A\beta$ deposition between EOAD and LOAD subjects. We anticipate our essay to be a starting point in the investigation of the use of textural parameters extracted in [$^{18}$F]florbetaben PET as potential biomarkers for early diagnosis of EOAD patients, thus going beyond the traditional concepts of quantitative PET for the study of neurodegenerations.

## Methods

### Patient selection

All subjects gave their informed consent. The study was conducted in accordance with the Declaration of Helsinki, and the protocol was approved by the Ethics Committee of the Policlinic Tor Vergata (Project identification code 158/16).

We enrolled 43 newly diagnosed AD patients according to the NIA-AA criteria. All AD patients were found positive to $A\beta$ deposition. Moreover, 27 control group subjects (CG) were recruited among a group of patients with subjective cognitive decline. They did not show subsequent progression to mild cognitive impairment at follow-up (average 24 months) and were considered as the control group. All the control subjects were found negative to $A\beta$ deposition. Doubtful cases at the visual examination were further checked by an experienced nuclear medicine physician (A.C.) by means of semi-quantitative analysis. We excluded subjects corresponding to the following criteria:

1. Subjects with the isolated deficit and/or unmodified mini-mental state examination (MMSE) during revisits, with a Hachinski scale and radiological evidence of sub-cortical lesions;
2. Presence of neurological symptoms as dysfunction of the hypothalamus and/or appendices suprasphenoidalis disease;
3. Presence of pyramidal and/or extrapyramidal signs at the neurological examination;
4. Presence of chronic illnesses: thyroid disease, diabetes, HIV, cancer, or previous brain injury.

We report the basic information of the complete dataset in Table 1.

**Table 1:** Overview of the subjects included in the study

	EOAD	LOAD	Control (age $<65$)	Control (age $\geq65$)
	[$n=14$]	[$n=29$]	[$n=9$]	[$n=18$]
Age (mean ± SD)	$58.93\pm 3.00$	$70.76\pm 4.28$	$60.89\pm 2.62$	$72.28\pm 3.71$
Gender (Woman; Man)	10 W; 4 M	16 W; 13 M	7 W; 2 M	13 W; 5 M
MMSE (mean ± SD)	$15.77\pm 6.56$	$18.82\pm 5.45$	–	–

### Scanning protocol

We conducted PET/CT scans in the Nuclear Medicine Facility of the Policlinic Tor Vergata, Rome, Italy. The Siemens Biograph PET/CT system at Tor Vergata was used to assess [$^{18}$F]florbetaben uptake.

All the subjects were injected intravenously with 295–320 MBq [$^{18}$F]florbetaben and hydrated with 500 ml of saline (0.9% sodium chloride). PET/CT scan was started approximately 90 min after [$^{18}$F]florbetaben injection. To avoid movement artifacts, four stacks of dynamic images (4 frames of 300 s each) were acquired in a 3D-mode standard technique.

The voxel size of PET image was $2.0\times 2.0\times 2.0$ mm$^3$. PET image reconstruction was performed using the three-dimensional ordered-subsets expectation maximization (OSEM) method with 4 subsets and 14 iterations.

### Image processing

**Fig. 1:**
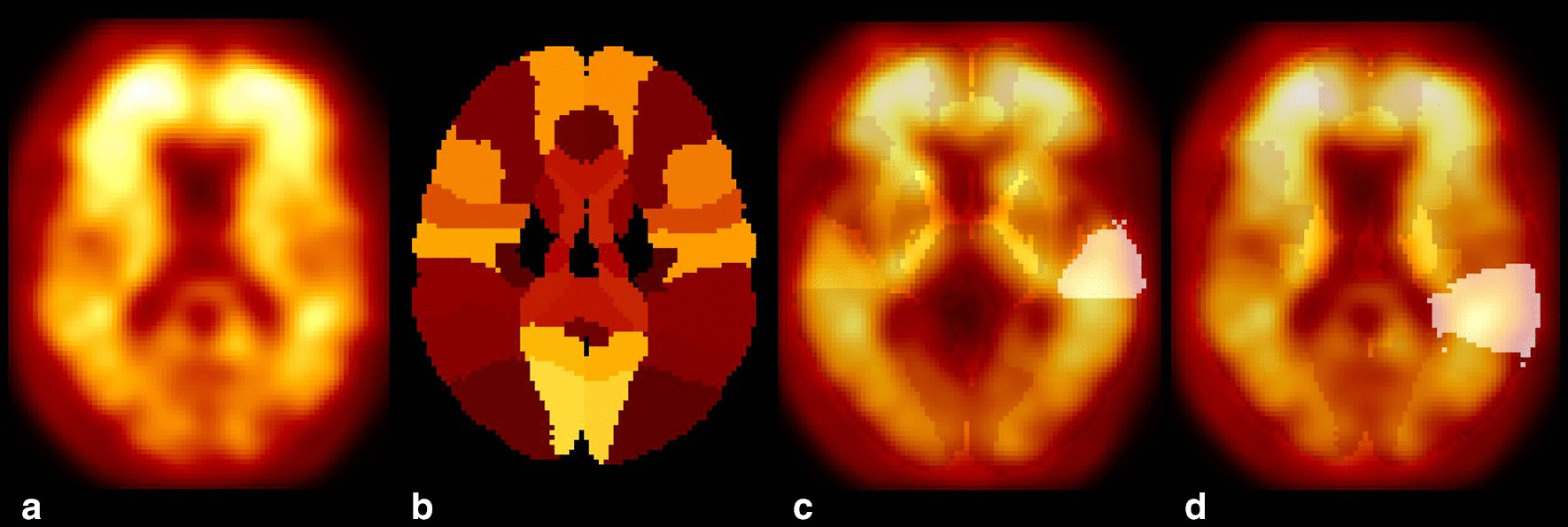
Registration of PET image and Hammers N30R83 Atlas. First, **a** the NIFTI PET image is normalized to the standard template (standard space composed of $91\times 109\times 91$ voxels with a resolution of $2\times 2\times 2$ mm$^3$). Second, **b** the Hammers N30R83 Atlas is normalized to the standard template space. Third, the VOIs are further extracted. As an example, the superior temporal gyrus **c** and the parietal gyrus **d** are shown

We performed the PET image registration and normalization steps by using statistical parametric mapping (SPM12). We first converted each individual [$^{18}$F]florbetaben PET brain DICOM image to Neuroimaging Informatics Technology Initiative (NIFTI) data. We then normalized the obtained NIFTI images to the standard International Consortium for Brain Mapping (ICBM) template using the mutual information affine registration (standard space composed of $91\times 109\times 91$ voxels with a resolution of $2\times 2\times 2$ mm$^3$) as in Fig. 1a. We resliced the Hammers N30R83 Atlas [25, 26] to the standard ICBM template using the mutual information affine registration with a resolution of $2\times 2\times 2$ mm$^3$ as reported in Fig. 1b. We identified each of the 83 Hammers areas as volume of interests (VOIs) with a point multiplication between the masks and the normalized PET images. For instance, we report the example of the superior temporal gyrus and the parietal gyrus in Fig. 1c, d, respectively.

We represented the Standardized Uptake Value (SUV) in each region using 256 gray levels. We further considered a rebinning with bin width $W=1,2,8,16,32,64,128$. For each VOI and each bin width, we calculated 93 textural features by using the software framework *pyradiomics (v3.0.1)* [27]. The textural parameters are extracted from six classes of matrices. The gray-level co-occurrence matrix (GLCM) quantifies the frequency of co-occurrence of a certain gray level within 26-connected neighbors of each pixel of the PET image [28, 29]. The gray-level dependence matrix (GLDM) quantifies gray-level dependencies in an image, and these gray-level dependencies quantify the distribution frequency of the 26-connected neighbors dependent on the center voxels [30]. To quantify the spatial distribution of adjacent gray levels, the gray-level run length matrix (GLRLM) textural features are used to represent the number of runs of a given length for each gray level [29, 31]. To identify structures with a certain tracer uptake, the gray-level size zone matrix (GLSZM) is used to classify the size of 3D volume with a given gray level [29]. Finally, in order to identify proper border features in a certain brain region, the neighborhood gray-tone difference matrix (NGTDM) is used to represent the sum of the gray-level differences between each pixel and the 26-connected neighbors [29, 32]. We report the list of studied parameters in Table 2.

**Table 2:** Textural parameters used in the analysis

Type	ID	Name	Width
GLDM	1	Dependence entropy	64
	2	Dependence non uniformity	64
	3	Dependence non uniformity normalized	64
	4	Dependence variance	64
	5	Gray level non uniformity	64
	6	Gray level variance	64
	7	High gray level emphasis	32
	8	Large dependence emphasis	64
	9	Large dependence high gray level emphasis	32
	10	Large dependence low gray level emphasis	32
	11	Low gray level emphasis	64
	12	Small dependence emphasis	1
	13	Small dependence high gray level emphasis	64
	14	Small dependence low gray level emphasis	64
GLSZM	15	Gray level non uniformity	1
	16	Gray level non uniformity normalized	64
	17	Gray level variance	64
	18	High gray level zone emphasis	64
	19	Large area emphasis	1
	20	Large area high gray level emphasis	8
	21	Large area low gray level emphasis	2
	22	Low gray level zone emphasis	64
	23	Size zone non uniformity	1
	24	Size zone non uniformity normalized	1
	25	Small area emphasis	1
	26	Small area high gray level emphasis	64
	27	Small area low gray level emphasis	8
	28	Zone entropy	2
	29	Zone percentage	1
	30	Zone variance	64
GLCM	31	Autocorrelation	32
	32	Cluster prominence	128
	33	Cluster shade	128
	34	Cluster tendency	64
	35	Contrast	128
	36	Correlation	64
	37	Difference average	64
	38	Difference entropy	64
	39	Difference variance	128
	40	Id	64
	41	Idm	64
	42	Idmn	64
	43	Idn	64
	44	Imc1	8
	45	Imc2	64
	46	Inverse variance	64
	47	Joint average	32
	48	Joint energy	64
	49	Joint entropy	64
	50	MCC	64
	51	Maximum probability	32
	52	Sum average	32
	53	Sum entropy	64
	54	Sum squares	64
NGTDM	55	Busyness	64
	56	Coarseness	64
	57	Complexity	128
	58	Contrast	8
	59	Strength	64
GLRLM	60	Gray level non uniformity	64
	61	Gray level non unifromity normalized	64
	62	Gray level variance	64
	63	High gray level run emphasis	32
	64	Long run emphasis	64
	65	Long run high gray level emphasis	64
	66	Long run low gray level emphasis	32
	67	Low gray level run emphasis	64
	68	Run entropy	64
	69	Run length non uniformity	64
	70	Run length non uniformity normalized	64
	71	Run percentage	64
	72	Run variance	64
	73	Short run emphasis	64
	74	Short run high gray level emphasis	64
	75	Short run low gray level emphasis	32
First order	76	10 Percentile	1
	77	90 Percentile	1
	78	Energy	1
	79	Entropy	1
	80	Entropy	1
	81	Kurtosis	1
	82	Mean absolute deviation	1
	83	Mean	1
	84	Median	1
	85	Robust mean absolute deviation	1
	86	Root mean squared	1
	87	Skewness	1
	88	Total energy	1
	89	Uniformity	64
	90	Variance	1
	91	Maximum	1
	92	Minimum	1
	93	Range	1

The last column reports the optimal bin width used for the estimate of each parameter

Although control subjects were found negative to $A\beta$ deposition, the background tracer perfusion is visible in the PET images. Therefore, the textural analysis of the [$^{18}$F]florbetaben PET of the control group provides a measurement of the textural parameters level of the background.

### Statistical analysis

The first step of the statistical analysis consisted of selecting the proper bin width for each textural parameter. To this aim, we calculated the correlation of the textural parameters and the age of onset in the AD patients group for each bin width by using a Spearman correlation statistical test. We selected the optimal bin width for which the Spearman correlation coefficient was maximal. At this stage, we remained 7719 parameters describing the textural properties of the [$^{18}$F]florbetaben uptake distribution in the brain, namely 93 textural features for each of the 83 Hammers regions.

The second step of the statistical analysis consisted of applying a features reduction strategy based on the principal components analysis (PCA). We identified in each brain region *r* the number $N_{{\rm ind}}^r$ of independent principal components $f_i^r$ accounting for 90% of the total variance [33, 34]. We restricted the further analysis to these independent principal components $f_i^r$. We studied the correlation between $f_i^r$ and the age of AD onset using the Spearman correlation statistical method, by selecting only those significant principal components $f_i^r$ exhibiting a correlation coefficient $|r|>0.5$ ($P<0.05/N_{{\rm ind}}^r$). We applied a Bonferroni correction taking into account the multiple comparisons between independent statistical tests within each brain region [35–38].

The third step of the statistical analysis consisted of identifying the age range at which EOAD and LOAD exhibit significant differences. To this aim, we considered the null hypothesis that EOAD and LOAD do not differ, where EOAD and LOAD were defined with respect to a variable threshold age of onset ${\mathcal{A}}$. We divided the patients into two groups, with the age of onset higher and lower than ${\mathcal{A}}$. Within each group, we calculated the average $\mu _{i}$ and the variance $\sigma _{i}$ of each of the significant principal components $f_i^r$. The ${\mathcal{A}}$ was spanned from 55 to 75 years. For each ${\mathcal{A}}$ and for each selected principal component, we calculated the probability that the null hypothesis was satisfied with a 2-tailed Student’s* t*-test by comparing the mean $\mu _{i}$ and the variance $\sigma _i$ in the two groups. We indicated the *P*-value obtained with [$^{18}$F]florbetaben PET imaging for each ${\mathcal{A}}$ as $P_{{\rm FBB}}\left( {\mathcal{A}}\right)$. We considered a *P*-value $P_{{\rm FBB}}\left( {\mathcal{A}}\right) _{{\rm sig}} = 0.05/N_{{\rm ind}}^r$ as expressing a 95% confidence level on the rejection of the null hypothesis, by including the multiple comparisons correction. Finally, we considered the range of threshold age of onset ${\mathcal{A}}$ of which the *P*-value was lower than $P_{{\rm FBB}}\left( {\mathcal{A}}\right) _{{\rm sig}}$ as the range of age of onsets for which EOAD and LOAD are significantly different at a 95% confidence level.

## Results

### Correlation of [$^{18}$F]florbetaben SUV and AD age of onset

**Fig. 2:**
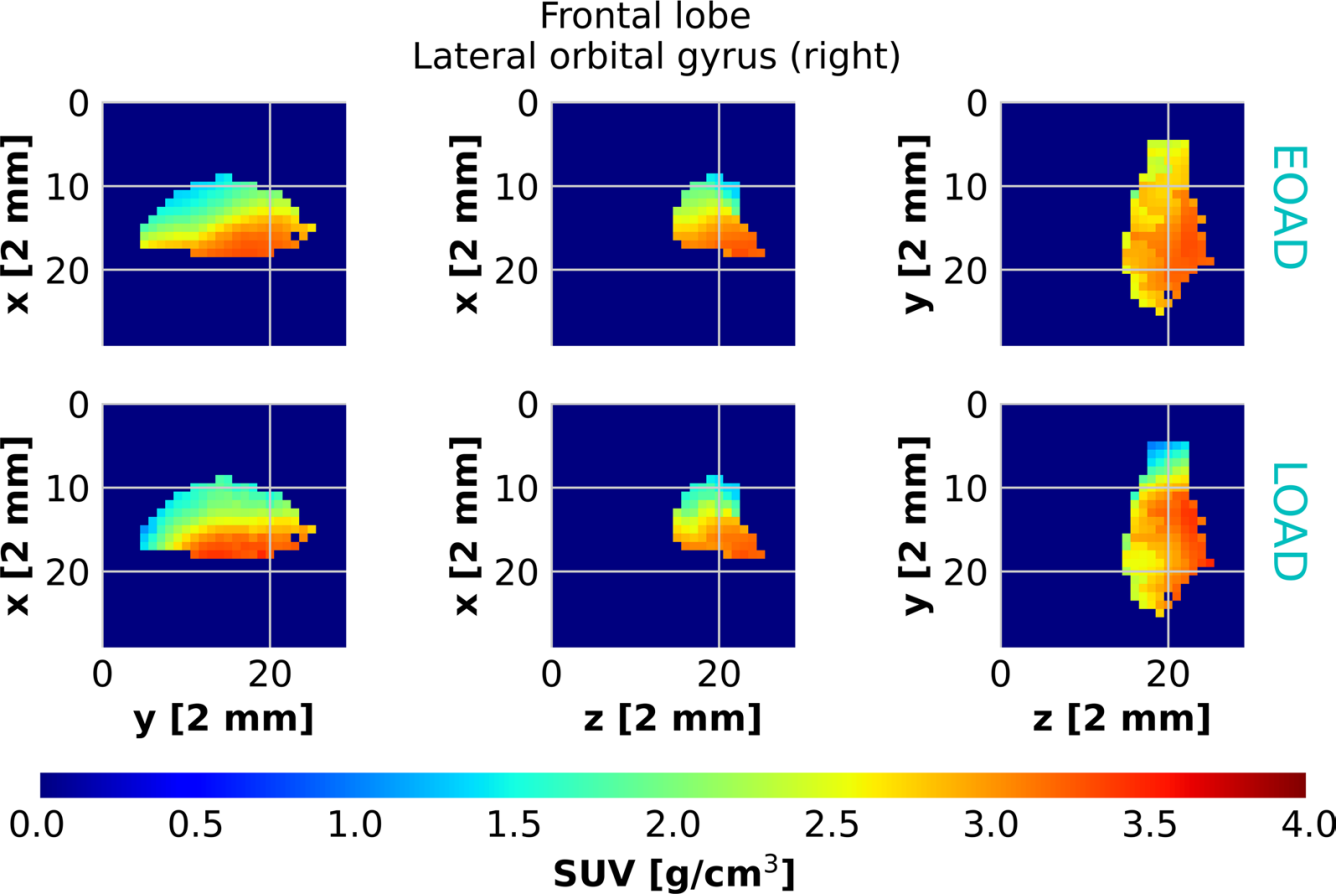
$A\beta$ plaques in the right lateral orbital gyrus of the frontal lobe, observed with [$^{18}$F]florbetaben PET. The right lateral orbital gyrus of the frontal lobe: maximal projection [$^{18}$F]florbetaben PET images of EOAD (upper row) and LOAD (lower row) patients. The SUV does not exhibit any significant difference between the two classes of patients

**Fig. 3:**
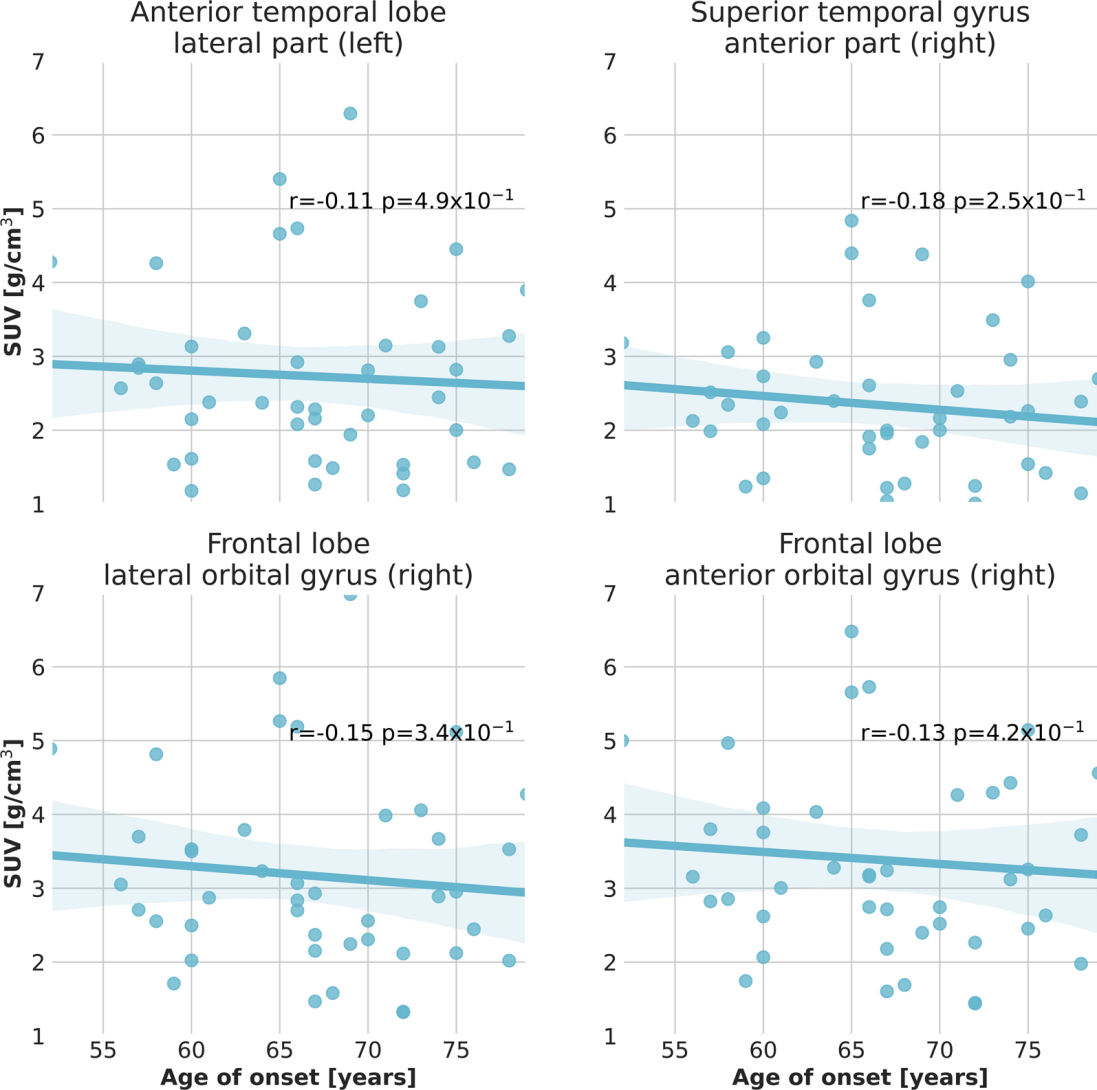
Average SUV in the temporal lobe and the frontal lobe observed with [$^{18}$F]florbetaben PET. The average SUV of [$^{18}$F]florbetaben uptake does not exhibit any correlation with the age of onset of AD. A linear regression with 95% confidence bands is shown on the plots for better visualization of the correlations

The SUV of [$^{18}$F]florbetaben uptake in the different Hammers brain areas of patients with AD diagnosis does not exhibit a dependence on the age of onset of the disease. As an example, we report the maximal projection [$^{18}$F]florbetaben PET image of the right lateral orbital gyrus of the frontal lobe for an EOAD and a LOAD patient in Fig. 2. As we observe in the figure, the average [$^{18}$F]florbetaben PET uptake does not significantly differ from EOAD and LOAD patients. To support this qualitative statement, we studied the dependence of the average [$^{18}$F]florbetaben PET uptake versus AD age of onset for the entire cohort of patients. We report the example of the left lateral part of the anterior temporal lobe, the right anterior orbital gyrus of the frontal lobe, the right lateral orbital gyrus of the frontal lobe and the left anterior part of the superior temporal gyrus in Fig. 3. We did not find any significant correlation between the average SUV and the age of onset of AD. We confirmed the absence of the correlation of average [$^{18}$F]florbetaben PET SUV in all the Hammers regions. Similarly, the Standard Uptake Value Ratio (SUVR) calculated by normalizing the value of the SUV to the background estimated in the amygdala did not exhibit any correlation with the age of onset.

### Correlation of textural features and AD age of onset

Despite the absence of a direct correlation of average [$^{18}$F]florbetaben PET uptake and age of onset of AD, a careful analysis of the 83 Hammers regions allows identifying heterogeneous structures and aggregates. This observation was at the basis of a quantitative characterization of these structures by means of textural features. We report the optimal bin width used for the estimation of the 93 textural parameters in each of the 83 Hammers regions in Table 2.

The textural parameters considered in this study are intimately correlated within each brain region, due to the fact that they are calculated on the basis of a finite set of six textural matrices. We performed a principal components analysis of the textural features in each brain region. We report the number of independent principal components accounting for 90% of the total variance in each region in Table 3.

**Table 3:** Brain regions and number of principal components

Region	ID	${N_{{\rm ind}}^r}$
*Frontal lobe*		
Middle frontal gyrus	28, 29	6, 5
Precentral gyrus	50, 51	6, 7
Straight gyrus	52, 53	6, 6
Anterior orbital gyrus	54, 55	7, 6
Inferior frontal gyrus	56, 57	6, 7
Superior frontal gyrus	58, 59	6, 7
Medial orbital gyrus	68, 69	8, 4
Lateral orbital gyrus	70, 71	6, 5
Posterior orbital gyrus	72, 73	6, 6
Subgenual frontal cortex	76, 77	7, 7
Subcallosal area	78, 79	8, 8
Pre-subgenual frontal cortex	80, 81	6, 7
*Temporal lobe*		
Hippocampus	2, 1	8, 7
Amygdala	4, 3	7, 8
Anterior temporal lobe medial part	6, 5	7, 7
Anterior temporal lobe lateral part	8, 7	6, 5
Parahippocampal and ambient gyri	10, 9	7, 8
Superior temporal gyrus posterior part	12, 11	5, 7
Middle and interior temporal gyrus	14, 13	6, 6
Fusiform gyrus	16, 15	6, 8
Posterior temporal lobe	30, 31	5, 7
Superior temporal gyrus anterior part	82, 83	7, 8
*Parietal lobe*		
Parietal gyrus	60, 61	7, 7
Superior parietal gyrus	62, 63	6, 8
Inferolateral remainder of parietal lobe	32, 33	6, 5
*Occipital lobe*		
Lateral remainder of occipital lobe	22, 23	6, 6
Lingua gyrus	64, 65	7, 6
Cuneus	66, 67	8, 8
*Central structure*		
Caudate nucleus	34, 35	8, 8
Nucleus accumbens	36, 37	8, 8
Putamen	38, 39	6, 8
Thalamus	40, 41	7, 7
Pallidum	42, 43	7, 6
Substantia nigra	74, 75	8, 7
Corpus Callosum	44	6
*Insula and cingulate gyri*		
Insula	20, 21	5, 6
Cingulate gyrus anterior part	24, 25	7, 9
Cingulate gyrus posterior part	26, 27	8, 7
*Posterior fossa*		
Cerebellum	18, 17	4, 5
Brainstem	19	5
*Ventricles*		
Lateral ventricle	46, 45	8, 8
Lateral ventricle temporal horn	48, 47	10, 5
Third ventricle	49	9

**Fig. 4:**
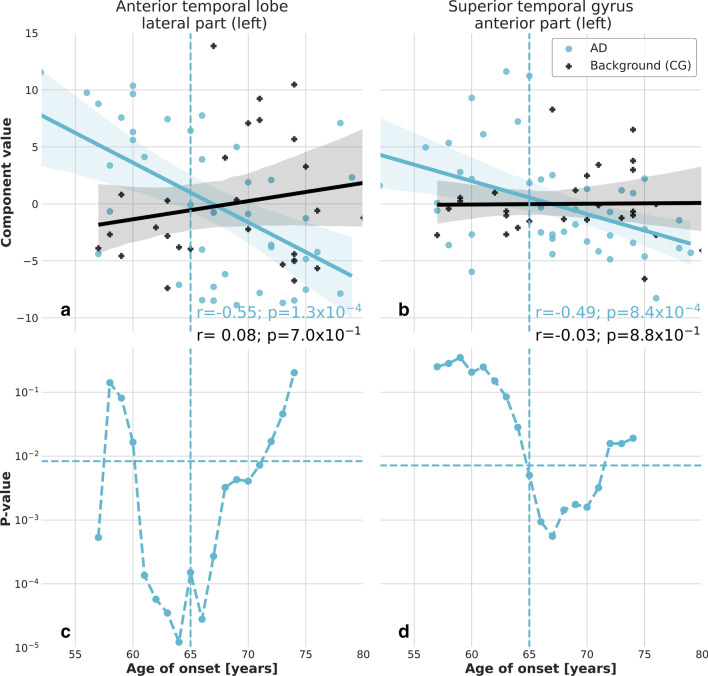
Correlation of the significant principal components and age of onset of AD in the temporal lobe. Principal components exhibiting significant correlation with the age of onset of AD $\left( |r|>0.5; P<0.05/N_{{{\rm ind}}}^r\right)$. A linear regression with 95% confidence bands is shown on the plots for better visualization of the correlations. The features extracted from AD patients in the left lateral part of the anterior temporal lobe (**a**) and in the left anterior part of the superior temporal gyrus (**b**) exhibit a significant difference with respect to the background estimated from control subjects, as confirmed also by the Student’s* t*-test on the distributions of the two patient categories with respect to a threshold age ${\mathcal{A}}$ (**c**, **d**)

**Fig. 5:**
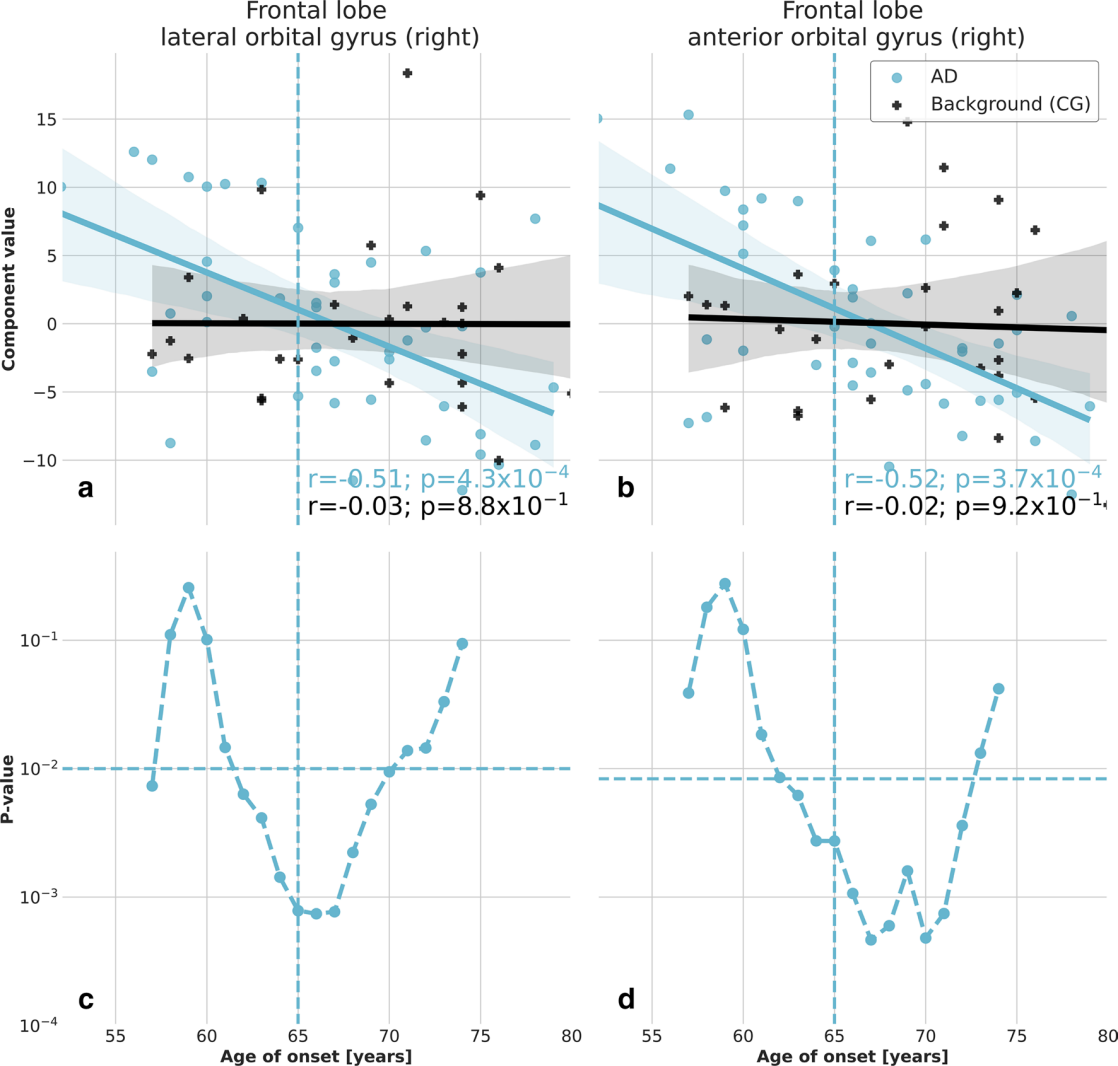
Correlation of the significant principal components and age of onset of AD in the frontal lobe. Principal components exhibiting significant correlation with the age of onset of AD $\left( |r|>0.5; P<0.05/N_{{{\rm ind}}}^r\right)$. A linear regression with 95% confidence bands is shown on the plots for better visualization of the correlations. The features extracted from AD patients in the right lateral orbital gyrus of the frontal lobe (**a**) and in the right anterior orbital gyrus of the frontal lobe (**b**) exhibit a significant difference with respect to the background estimated from control subjects, as confirmed also by the Student’s *t*-test on the distributions of the two patient categories with respect to a threshold age ${\mathcal{A}}$ (**c**, **d**).

We found that four principal components exhibit a strong correlation with the age of onset of AD in the temporal lobe (Fig. 4a, b) and in the frontal lobe (Fig. 5a, b), respectively. The component $f_{0}$ in the left lateral part of the anterior temporal lobe accounts for 43.7% of the total variance and exhibits a significant correlation with the age of onset of AD (*r* = − 0.55, *p* = $1.3\times 10^{-4}$). The background estimated using control subject data does not exhibit any significant correlation. Similarly, the component $f_0$ in the right anterior orbital gyrus of the frontal lobe accounts for 47.2% of the total variance and exhibits a significant correlation with the age of onset of AD (*r* = − 0.52, *p* = $3.7\times 10^{-4}$). The component $f_0$ in the right lateral orbital gyrus of the frontal lobe accounts for 50.8% of the total variance and exhibits a significant correlation with the age of onset of AD (*r* = − 0.51, *r* = $4.3\times 10^{-4}$). The component $f_1$ in the left anterior part of the superior temporal gyrus accounts for 21.0% of the total variance and exhibits a significant correlation with the age of onset of AD (*r* = − 0.49, *r* = $8.4\times 10^{-4}$). We report the main features contributing to the linear expansion of the four principal components in Table 4. They are identified as the ones with coefficients $\left| c_j\right| > 0.9\times \max \left| c_i\right|$.

**Table 4:** Selected principal components and most significant textural features

Principal component	Region	Textural features
$f_0$	Left lateral part of the anterior temporal lobe (8)	1, 2, 3, 5, 6, 8, 32, 34, 35, 37, 38, 39, 40, 41, 42, 43, 45, 46, 48, 49, 53, 54, 57, 58, 60, 61, 63, 64, 69, 70, 71, 79, 92
$f_0$	Right anterior orbital gyrus of the frontal lobe (55)	1, 5, 6, 12, 16, 29, 30, 34, 49, 53, 54, 56, 60, 61, 79, 80, 83, 88, 92
$f_0$	Right lateral orbital gyrus of the frontal lobe (71)	2, 3, 5, 6, 8, 32, 35, 37, 38, 39, 40, 41, 42, 43, 44, 45, 46, 48, 49, 53, 54, 60, 61, 62, 64, 68, 69, 70, 71, 79, 83, 92, 93
$f_1$	Left anterior part of the superior temporal gyrus (82)	7, 9, 11, 31, 47, 52, 63, 65, 67, 76, 78, 84, 85, 86, 89, 91

### Difference between EOAD and LOAD

**Fig. 6:**
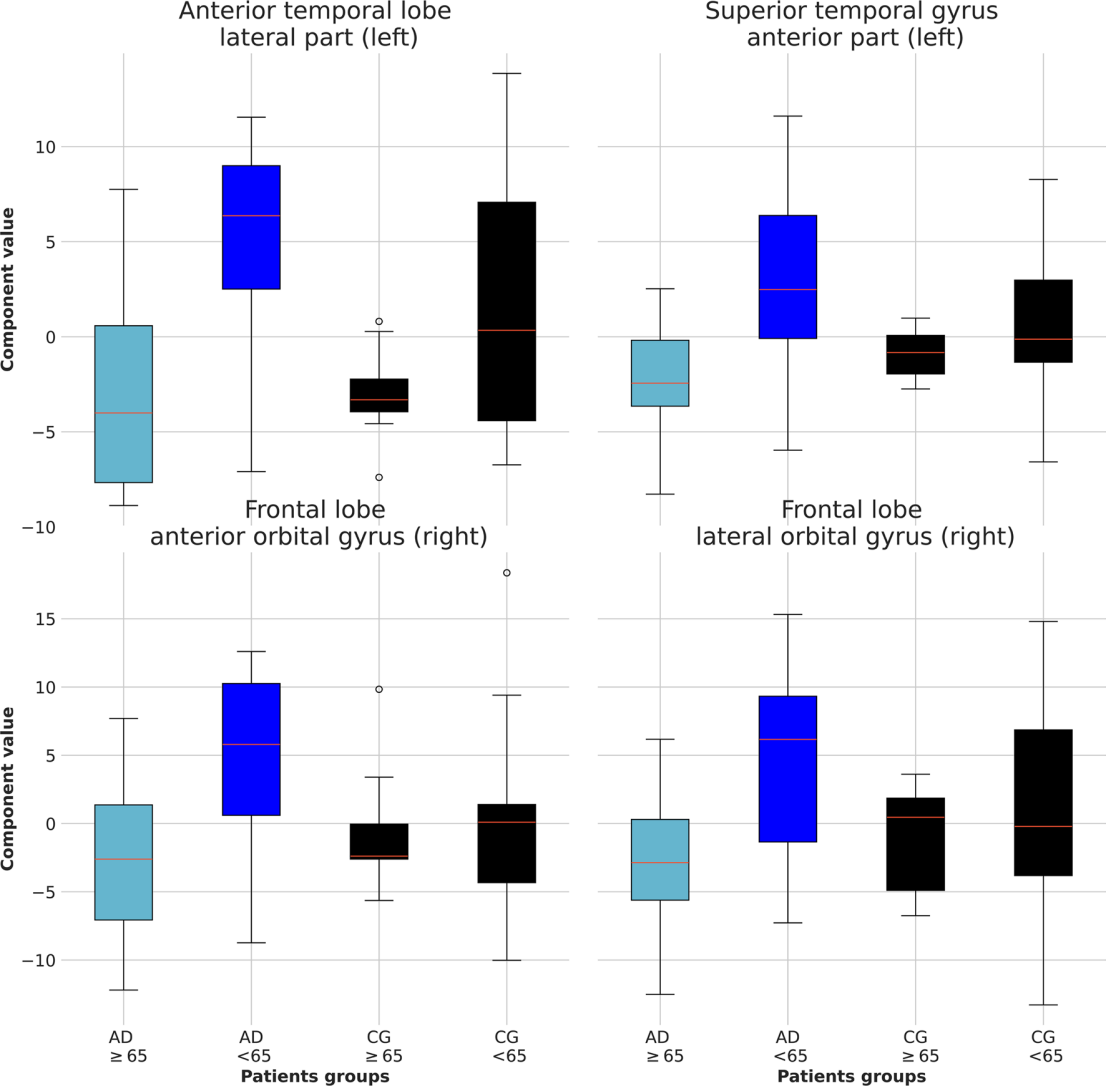
Candle plot in the temporal lobe and the frontal lobe for LOAD, EOAD, and background estimated from control subjects. Candle plots of the distribution of the significant principal components for LOAD, EOAD, and background from control subjects. The age of onset of AD is classified according to the threshold age *A* = 65. The average value of the parameters for EOAD patients exhibits difference with respect to LOAD and background estimated from control subjects, in support to the assumption that the identified principal components describe intrinsic early-onset features of the spatial distribution of the $A\beta$ deposition in [$^{18}$F]florbetaben PET

The correlation of the four principal components identified above and the age of onset of AD can be used to clarify the threshold age ${\mathcal{A}}$, at which the group of EOAD and LOAD patients is significantly different. In order to provide a graphical representation of this concept, we show the candle plot of the value of the significant components for AD and control subjects with a threshold age ${\mathcal{A}}=65$ years in Fig. 6. We observe that the value of the textural parameters in EOAD patients differs of approximately 20% between EOAD, LOAD and background uptake estimated from control subjects.

For a more quantitative estimation of the level of significance, we report in Figs. 4c, d and 5c, d the *P*-value of the Student’s *t*-test applied to the distribution of the four principal components in the left lateral part of the anterior temporal lobe, the right anterior orbital gyrus of the frontal lobe, the right lateral orbital gyrus of the frontal lobe and the left anterior part of the superior temporal gyrus corresponding to EOAD and LOAD patients defined with an age of onset threshold ${\mathcal{A}}$. Parameter ranges can be identified for those principal components, in which a 95% confidence level significance is reached, corresponding to the age range for which $P<0.05/N_{{\rm ind}}^r$. In particular, the four identified principal components exhibit a significant difference between the EOAD and LOAD groups, when the threshold age is set at 65 years.

## Discussion

The findings presented above need to be discussed from the perspective of both medical physics and clinical significance. The key advantage of the textural analysis approach used in this paper is the possibility to identify a clear signature of EOAD patients. We studied the $A\beta$ deposition measured with [$^{18}$F]florbetaben PET in 83 Hammers brain regions, and despite the absence of a significant correlation of any first-order statistical characteristics of the SUV distribution and age of onset of AD, we found four independent principal components in the left lateral part of the anterior temporal lobe, the right anterior orbital gyrus of the frontal lobe, the right lateral orbital gyrus of the frontal lobe and the left anterior part of the superior temporal gyrus exhibiting a significant correlation with the age of onset of AD.

The features generating the principal components reflect the degree of association between different voxels in the same brain region [32]. By way of example, the GLDM describes the gray-level dependencies in an image. A higher value of the textural features extracted from GLDM implies a larger number of areas with different sizes and different values. In other words, a larger value of a textural feature extracted from GLDM corresponds to a heterogeneous and irregular spatial distribution, with a series of complex correlation structures between the voxels. The textural analysis proposed in this paper suggests in particular that the $A\beta$ deposition in the left lateral part of the anterior temporal lobe, the right anterior orbital gyrus of the frontal lobe, the right lateral orbital gyrus of the frontal lobe and the left anterior part of the superior temporal gyrus follows a significantly more complex pattern in correspondence to an early age of onset of AD.

The use of the textural features for the verification of the presence of intrinsic structures and patterns finds agreement in other studies [22]. In particular, textural features extracted from GLDM were relevant to cognitive scale values and correlated with the insurgence of AD and MCI, suggesting that 2-[$^{18}$F]FDG brain PET imaging not only can be used for classification diagnosis but also contain information related to the pathological process [39–42]. Abnormalities of the pattern of $A\beta$ deposition were also observed in connection to AD. Textural features extracted from the GLRLM in the fused [$^{18}$F]florbetaben PET/MRI images were also used recently to understand cognitive and behavioral issues in patients with AD [22]. Textural features of MRI images were also used for the identification of structural and morphological alterations occurring in the hippocampus and in the corpus callosum of AD and MCI subjects, as well as of patients with autism spectrum and hyperactivity disorder [20, 21, 43–45]. A limitation of previous studies, as noticed also in [22], is the absence of an age-dependent classification. Our study is thus showing that such pre-classification is not only needed, but also reveals a fundamental difference between patients with an early and a late diagnosis of AD. The analysis of $A\beta$ deposition data represents also a unique point of innovation of this paper with respect to previous findings.

**Fig. 7:**
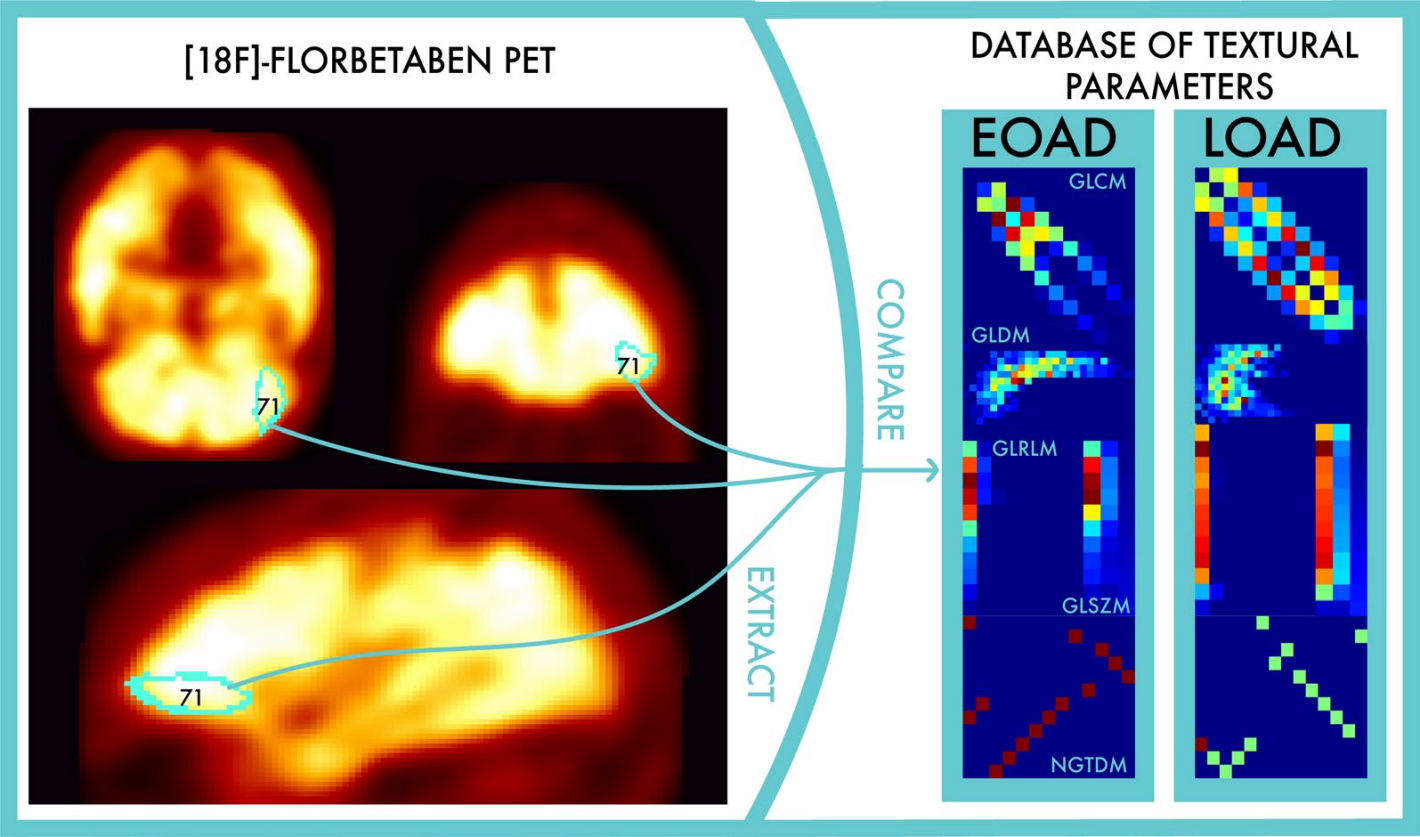
Example of analysis in the lateral orbital gyrus, frontal lobe. Example of EOAD verification: the three-dimensional ROI corresponding to the right lateral orbital gyrus in the frontal lobe is extracted from the [$^{18}$F]florbetaben PET images, the textural parameters are calculated and compared with the database of patients collected in this paper, identifying EOAD candidates

Our observation is consistent with most findings reported in the literature for the characterization of AD patients [19, 46–50]. More interestingly, our findings stress the evidence of differences between EOAD and LOAD, thus indicating a possible intrinsic pathological ground. The statistical methods applied in this study in fact justify a difference of the distribution pattern of the $A\beta$ deposition in the left lateral part of the anterior temporal lobe, the right anterior orbital gyrus of the frontal lobe, the right lateral orbital gyrus of the frontal lobe and the left anterior part of the superior temporal gyrus. In this paper, we developed a prognostic model, schematically represented in Fig. 7, which is ready to be externally validated on independent datasets and independent clinical trials [51]. However, more gender-differentiated data would be needed to understand whether epidemiological factors play a role in this analysis. Moreover, larger statistics may enhance the significance of the brain regions that, although exhibiting an age-dependent behavior, do not disentangle EOAD and LOAD patients.

In a recently published study, we reported that the increased $A\beta$ deposition alone cannot explain the clinical differences between EOAD and LOAD patients [17]. The results presented here are not in contradiction with previous findings. While in [17] we made the observations only based on the SUV, here we extended the analysis to the recognition and possible interpretation of patterns. Different metabolic patterns between EOAD and LOAD were identified in the left parietal lobe and in inferior brain regions by using 2-[$^{18}$F]FDG PET [52, 53], contributing to the hypothesis that the regional vulnerability of associative areas of the brain represents a possible signature of EOAD. Moreover, extrapyramidal symptoms and frontal dysfunction are more common in EOAD than in LOAD patients [53], therefore supporting our observations in the left lateral part of the anterior temporal lobe, the right anterior orbital gyrus of the frontal lobe, the right lateral orbital gyrus of the frontal lobe and the left anterior part of the superior temporal gyrus. The role of $A\beta$ in this scenario, however, is still unclear. Our findings enhanced the potential association of a highly fragmented $A\beta$ distribution with the age of onset of AD. This observation allows a large number of possible explanations. For example, as already suggested in [17], the increased textural disorder observed in the $A\beta$ deposition and glucose metabolism may be in fact related to the inability to reduce highly toxic soluble $A\beta$ oligomers into relatively less toxic insoluble plaques.

## Conclusions

We have found that four principal components exhibit a significant correlation at a 95% confidence level with the age of onset in the left lateral part of the anterior temporal lobe, the right anterior orbital gyrus of the frontal lobe, the right lateral orbital gyrus of the frontal lobe and the left anterior part of the superior temporal gyrus. Data are consistent with the hypothesis that AD patients with age of onset at the age younger than 65 years exhibit significant difference with respect to patients with later onset at 95% confidence level in the spatial patterns of the $A\beta$ deposition in the left lateral part of the anterior temporal lobe, the right anterior orbital gyrus of the frontal lobe, the right lateral orbital gyrus of the frontal lobe and the left anterior part of the superior temporal gyrus.

Our findings support the textural analysis approach to the investigation of the AD pathology, intending to define novel biomarkers for a faster clinical diagnosis of EOAD. The textural parameters identified in this paper are the basis of further experiments in order to investigate the biological and functional interpretation of the mechanism on the basis of this typical EOAD signature.

## Data Availability

The datasets generated during and/or analyzed during the current study are available from the corresponding author on a reasonable request.

## Abbreviations

AD: Alzheimer’s disease
$$A\beta$$: Amyloid-$$\beta$$
DICOM: Digital imaging and communications in medicine
EOAD: Early-onset AD
FBB: [$$^{18}$$F]florbetaben
2-[$^{18}$F]FDG: 2-deoxy-2-[$^{18}$F]fluoro-d-glucose
GLCM: Gray-level co-occurrence matrix
GLDM: Gray-level dependence matrix
GLRLM: Gray-level run length matrix
GLSZM: Gray-level size zone matrix
HC: Healthy control
CG: Control group subjects
ICBM: International Consortium for Brain Mapping
LOAD: Late-onset AD
MCI: Mental cognitive impairment
MMSE: Mini-mental state examination
MRI: Magnetic resonance imaging
NGTDM: Neighborhood gray tone difference matrix
NIFTI: Neuroimaging informatics technology initiative
PCA: Principal components analysis
PET: Positron emission tomography
SPM: Statistical parametric mapping
SUV: Standardized uptake value
SUVR: Standard uptake value ratio
VOIs: Volume of interests
